# Risky sexual behavior and its associated factors among patients with severe mental disorder in University of Gondar Comprehensive Specialized Hospital, 2018

**DOI:** 10.1186/s12888-021-03054-z

**Published:** 2021-01-21

**Authors:** Daniel Ayelegne Gebeyehu, Missaye Mulatie

**Affiliations:** 1 Department of Community Health Nursing, School of Nursing, College of Medicine and Health Sciences, University of Gondar, Gondar, Ethiopia; 2 Department of Psychology, College of Social Sciences and the Humanities, University of Gondar, Gondar, Ethiopia

**Keywords:** Risky sexual behavior, Severe mental disorder, Gondar, Ethiopia

## Abstract

**Background:**

People with severe mental disorders are more likely to engage in high-risk sexual behaviors. As a result of these high-risk behaviors, they might contract sexually transmitted infections and become pregnant unintentionally. Despite the high burden of this problem, very little is known about the association between mental disorders and high-risk sexual behaviors; for this reason, the current study aimed at determining the association between these two behaviors in patients with mental disorders attending an outpatient clinic at the University of Gondar Comprehensive Specialized Hospital, Psychiatric Clinic.

**Methods:**

A total of 223 study participants were recruited via a stratified sampling followed by a systematic sampling technique. An institutional-based cross-sectional study was conducted from April to May 2018. Data were collected using a pretested interviewer-based questionnaire. A four-item questionnaire was adopted from a behavioral surveillance survey and different literature sources. A multivariable logistic regression model was fitted to assess the strength in addition to the direction of the association between risky sexual behavior and independent variables. An interpretation was made based on the adjusted odds ratio and *p*-value at a 95% confidence interval (CI).

**Result:**

Nearly half of the study participants (49.8, 95% CI; 43.9–56.5%) presented risky sexual behavior. The study found that male gender (2.98; adjusted odds ration [AOR] = 2.98; 95% CI; 1.49–5.95), no ability to read and write (3.99; AOR = 3.99; 95% CI: 1.53–10.4), history of hospitalization (3.95; AOR = 3.95; 95% CI: 1.87–8.32), perceived internal stigma (2.45, AOR = 2.45; 95% CI: 1.18–5.11), and poor social support (3.07, AOR = 3.07; 95% CI: 1.29–7.30) were significant predictors of risky sexual behaviors among patients with severe mental disorder.

**Conclusion:**

Risky sexual behavior among patients with a severe mental disorders was high (49.8%). Special attention should be given to male patients, incorporating people with severe mental disorders into the adult education programs, continuous health education regarding risky sexual behavior and utilization of condoms, building self-esteem, and engaging others to provide good social support systems are strongly recommended to alleviate this type of behavior in this population.

## Background

Risky Sexual Behavior (RSB) is an act that increases one’s risk of contracting sexually transmitted infections and experiencing unintended pregnancy [1]. It includes risky behaviors such as having multiple sexual partners, a history of unprotected sex/ failure to use condoms or intermittent use, exchanging money for sex, performing sexual intercourse while under the influence of alcohol [1, 2].

Psychiatric disorders may be more strongly associated with sexual risk Behaviors than others as a mental Disorder involves psychological problems that sway the individual’s cognition, emotional regulation, and behaviors [3]. Currently, Severe Mental Disorder (SMD) accounts for about 12% of the global burden of disease and is projected to reach 15% by the year 2020 [4].

Unsafe sex such as having sex without or failure to use a condom is ranked second among the top ten risk factors to health in terms of the burden of disease they cause [5, 6]. This magnitude is higher among patients with SMD; RSB among patients with SMD is amounted to be 66 and 34% among mood disorders and patients with schizophrenia in the United States of America, respectively [7]. In Brazil, about 83% of the participants engaged in RSB [8]. Moreover, Cross-sectional studies conducted in Zimbabwe, Turkey, and Nigeria have demonstrated that 23.8, 47.9, and 48% of patients with SMD experienced RSB, respectively [9–11].

As SMD affects the individual’s cognition, emotion, as well as behavior, patients with SMD are highly prone to performing unsafe sex [12–14] and more likely to develop sexually transmitted diseases, including, but not limited to, HIV infection [15], Hepatitis B and C, Herpes, Syphilis, and *Neisseria gonorrhoeae* [16–19]. The negative consequences may also include family conflicts, damage to relationships, legal disputes, and financial problems [20].

A number of factors contribute SMD patients to exercise RSB, for instance, younger age individuals are more prone to exercise RSB [10]. This might be due to the fact that these segments of the population are more impulsive, altered on judgment, sensitive to personal rejection, and low self-esteem [21]. As a result, they might have multiple sexual partners with acting sexual intercourse without a condom [22]. Unmarried individuals are also at risk for practicing RSB [23], as having multiple sexual partners is more prominent than married ones [24]. Patients in the acute phase of the disorder are more exposed to practicing RSB; this could be associated with the general impairment of reality testing and judgment that have commonly been seen amongst this population [25]. Poor social support and perceived internal stigma also have impacts on RSB; since good social support boosts one’s self-esteem and might help them in order to refrain from practicing risky behaviors [26–28]. Furthermore, substance use increases the risk of RSB by its disinheriting effect on one’s decision to engage in sexual behavior [29].

In Ethiopia, the magnitude and contributing factors of RSB among high school and college students, and adults who are ‘of the street’ or having “no home but the streets” were investigated [2, 30–32], frequently. Nevertheless, none of the previous studies showed that the magnitude and associated factors of RSB among patients with SMD, since the chance of practicing RSB are higher in this segment of the population when compared to the others [15]. In addition, it’s believed that some problems like poor social support and perceived internal stigma would have a contribution to practice RSB, but left uncovered.

Understanding the problem could help local decision-makers as it helps to design a comprehensive strategy to tackle the problem as early as possible. Furthermore, for clinicians, it might be helpful to prevent RSB or reduce the chances that their patients engage in potentially harmful sexual behaviors that adversely affect mental health outcomes by identifying the prevalence and factors that are associated with RSB. Likewise, for the clients; it helps to create awareness and tackling the determinants early. Therefore, the current study aimed at determining the magnitude of RSB and associated factors among patients with SMD, who were attending an outpatient clinic at University of Gondar Compressive Specialized Hospital (UOGCSH), Psychiatric clinic, June 2018.

## Methods

### The study area, design, and population

An institutional-based cross-sectional quantitative study design was conducted from April 02 to May 30, 2018. The study was carried out at the UOGCSH which is situated in the Northwest of Addis Ababa, the capital of Ethiopia. The hospital is found 750 km away from Addis Ababa. It is rendering services for about five million people [33]. The hospital provides inpatient and outpatient medical services in several departments including the Psychiatric Department. It has more than 1000 patient beds and provides service to over 200,000 patients annually [34]. The services provided to patients referred from other institutions including psychiatric care [35]. The hospital psychiatric clinic has four outpatient departments and four wards having 25 beds. For the purpose of this study, patients who were diagnosed with Schizophrenia, Major Depression, or Biopolar disorder, according to the DSM IV criteria, were considered as having SMD [36–39]. All patients diagnosed with SMD were considered as a source population, while those participants who were available during the data collection period were the study participants.

### Inclusion and exclusion criteria

Patients with 18 years old or more, diagnosed with Schizophrenia, MDD, or Bipolar disorder based on the DSM-IV [36–40], and that had two or more previous visits to the institution were included since patients who are in the first visit commonly have acute disturbance, which could affect the estimate of the study. Likewise, those patients who were acutely disturbed (participants in the active phase of illness) were excluded as they may lack insight and can’t respond to the questions properly.

### Sampling technique and procedure

The required sample size was estimated using a single population proportion formula by considering the following statistical assumptions; the prevalence of risky sexual behavior,50% (since there is no similar study conducted in the country among SMD patients) [41], 95% confidence interval, 5% degree of precision, and 10% non-response rate, which yielded a total sample size of 384. However, since our source population was less than 10,000, we have used the correction formula and the final sample size was 238.

The average number of SMD patients who visited the outpatient department in the past 1 month was estimated to be 500, of which about 190, 150, and 160 were schizophrenia, bipolar, and MDD, respectively. Then, participants were stratified based on their disease entity. Accordingly, proportional allocation was done to schizophrenia (*n* = 90), Bipolar (*n* = 72), and MDD (*n* = 76). The participant was selected every 2 intervals (K = 2) via the systematic sampling technique.

To get reliable data, patients’ insight towards the recognition of having a mental illness, notifying unusual behaviors as a problem/pathological, and the need for treatment was measured prior to selecting the study participants by mental health professionals (Those who had MSc. in Integrated Clinical and Community Mental Health). It was conducted by using three questions and 1 was coded as “Yes” and 0 for “No.” The total score ranged between 0 and 3. After the summation of the scores, the interpretation was made based on; a score of zero, 1–2, and 3 as in “no insight, “partial insight, and “full-insight, respectively.” Then, those participants who had full-insight were invited and enrolled in the study [42].

### Patient and public involvement

The outcome variable (RSB) was determined by using a four-item tool that was adapted from behavioral surveillance survey questions and other kinds of literature in Ethiopia after the pre-test was conducted. It was translated from the English language to the local language ‘Amharic’ by language experts. A total of 223 study participants who had full insight were recruited via stratified sampling followed by a systematic sampling technique. Written informed consent was obtained from the study participants. The findings of the study have been disseminated to the study participants through key stockholders, for instance; psychiatrists, and mental health professionals working in the psychiatry clinic.

### Data collection tools and procedures

The questionnaires were translated from the English language to Amharic (local language) by language experts and transcribed back to English to check the consistency and equivalence. The questionnaires were adapted from previously published articles and the tools were comprised of socio-demographic characteristics, measurements of substance use, and risk-related sexual information. A face-to-face interviewing method was used to collect the entire data.

RSB was determined by using a four-item tool which was adapted from behavioral surveillance survey questions and other literature in Ethiopia [2, 30, 43–45]. The questions were incorporating (1) the number of sexual partners, it was asking the individuals having two or more sexual partners, (2) history of unprotected sex, like incorrect use or failure to use a condom at least once during sexual intercourse until the survey; nevertheless, monogamous participants were excluded for this question, (3) Exchanging money for sex, like a sexual act with commercial sex workers, and (4) alcohol, or drug-driven unprotected sexual intercourse. Accordingly, if a participant reported at least one in the last year of the treatment period among the aforementioned questions, he/she labeled as practicing RSB.

The Oslo-3 Social Support Scale with three questions was used to measure the social support status of the study participants. Accordingly, participants who scored 12–14, 9-11and 3–8 were categorized as having good, moderate, and poor social support, respectively [46].

A 3-item tool on perceived stigma screening was used to assess the participants’ perceived stigma. Thus, a score of less than one indicates the absence of perceived stigma whereas a score of one or more indicates the presence of perceived stigma [47].

Regarding substance taken by the participants, at least any of the following substances: alcohol, Khat, and cigarette in the past 1 year of the treatment period that is assumed to affect the level of thinking and increase the risk of involving in risky sexual behaviors were taken into consideration [43].

To assure the quality of the data, a pre-test was conducted prior to the data collection on 5% of study participants at Bahir-Dar Felege Hiwot Hospital. The reliability test was performed for RSB questionnaires using Cronbach’s alpha and initially, the value was 0.62. After the deletion of one item of variance, it increased to 0.74. Training was given to data collectors regarding the techniques of interviewing, as well as filling the checklist for two-hours. Three diploma holder nurses for data collection and two Bachelor degree holders’ psychiatry nurses for supervision were recruited. Finally, the completeness of questionnaires was checked regularly.

### Data processing and analysis

Data were coded and entered into EPI info version 7 and exported into a Statistical Package for.

Social Science (IBM-SPSS) Version 21.0 for further analysis. The outcome variable, RSB was coded as ‘0’ and ‘1’ representing without and with the outcome, respectively. The findings of the study were presented using data summary measures like texts, tables, and figures. To identify the associated independent variables with RSB, the binary logistic regression analysis was employed. Accordingly, variables that had a *p*-value of ≤0.05 in the bi-variable analysis were entered into the multivariable regression model to control the effects of confounders as well as to identify the significant factors. Finally, variables having a *p*-value of ≤0.05 in multivariate analysis were considered as statistically significant; the interpretation of the data was made using the adjusted odds ratio and *p-*value with a 95% CI.

## Results

### Socio-demographic characteristics

A total of 223 patients were included in the study, attaining a response rate of 94%. Half (50.7%) of the study participants were females. The mean (±SD) age of the participants was 30(± 8.8) years. A bit greater than one-third (36.3%) of the participants was under the age category of 25 to 34 years. A large proportion (82.1%) of them was orthodox Christians. A bit lower than half (42.6%) of the participants were single (Table 1).

**Table 1:** Socio-demographic characteristics of the participants with severe mental disorders attending at the University of Gondar Comprehensive specialized referral, Hospital, 2018 (*n* = 223)

Variables	Frequency (n)	Percentage (%)
**Sex**		
Male	110	49.3
Female	113	50.7
**Age**		
18–24	71	31.8
25–34	81	36.3
35–44	51	22.9
≥ 45	20	9.0
**Religion**		
Orthodox	183	82.1
Muslim	30	13.5
Protestant*	10	4.4
**Marital status**		
Single	95	42.6
Married	69	30.9
Divorced	59	26.5
**Employment status**		
Employee	36	16.1
Private	37	16.6
Daily Worker	20	9.0
Unemployed	46	20.6
Student	62	27.8
Farmer	22	9.9
**Educational status**		
Can’t read and write	53	23.8
Primary (1-8grade)	54	24.2
Secondary (9–12)	59	26.5
Diploma and above	57	25.6

### Clinical and behavioral characteristics

Regarding the disease’s onset, two-thirds (139 (62.3%)) of the participants reported that the disorder begun before the age of 25-years-old. A bit more than half (120 (53.8%)) of the study participants stated that there has been less than 5-years since the illness has begun. A significant majority (70.9%) of the study participants had no history of hospitalization. Concerning the disorder, about 78 (35.0%), 69 (30.9%), and 76 (34.1%) of the participants were suffered from schizophrenia, Bipolar, and MDD, respectively.

Regarding substance use, a bit less than half (46.6%) of them used alcohol, and almost all (96.9%) participants haven’t smoked cigarettes. Furthermore, 31 (13.9%) of the participants had a history of chewing chat/khat in the last year of the treatment period.

### Patient /family-related factors

Slightly greater than two-thirds (67.7%) of the participants have perceived stigma. Around two in every ten (4.9%) of the respondents had no home. A small minority (4.9%) of them ever experienced sexual abuse (Table 2).

**Table 2:** the frequency of RSB and Patient/Family-related factors of a patient with severe mental disorder attending at University of Gondar Compressive Specialized Hospital, psychiatric department, 2018 (*n* = 223)

Variable	Frequency(n)	Percentage %
Risky Sexual Behavior		
Having sex without or failure to use a condom	59	53.2%
Having sex with two or more persons	34	30.6%
Alcohol driven sex	13	11.7%
Paid or received money for sex	5	4.5%
History of homelessness		
**Yes**	11	4.9
**No**	212	95.1
History of sexual abuse		
**Yes**	11	4.9
**No**	212	95.1
Perceived Internal stigma		
**Yes**	151	67.7
**No**	72	32.3
Social support		
**Good**	80	35.9
**Intermediate**	81	37.2
**Poor**	62	27.8

### Prevalence of risky sexual behavior

As shown in Table 2, close to half 49.8% (95%CI, 43.9, 56.5%) of the participants reported the experience of RSB. Among participants who had an experience of RSB, on the subject of the questions to measure risky sexual behavior, A bit greater than half (53.2%) of the respondents were reported, having sex without or failed to use a condom in the last year of the treatment period (Table 2).

### Factors associated with RSB

As shown in Table 3, after adjusting ORs, variables such as Sex, level of Education, type of disorder, history of hospitalization, Stigma, and social support were associated with practicing RSB (Table 3).

**Table 3:** Bi-variable and multivariable factors associated with RSB among patient with severe mental disorder attending at the University of Gondar Comprehensive Specialized referral Hospital, psychiatric department, 2018 (*n* = 223)

Explanatory variables	Risky Sexual Behavior		COR (95% CI)	AOR (95% CI)
	YES	NO		
Sex				
Male	63	47	1.81 (1.06,3.08)	2.98 (1.49,5.95)
Female	48	65	1	
Marital Status				
Married	26	43	1	1
Single	52	43	2.00 (1.06,3.76)	1.70 (0.78,3.68)
Divorced	33	26	2.00 (1.03,4.26)	1.78 (0.73,4.32)
Educational level				
Can’t read and write	28	25	1.92 (0.89, 4.11)	3.99 (1.53,10.4)
Grade 1–8	31	23	2.31 (1.07,4.95)	3.83 (1.49,9.79)
9–12	31	28	1.89 (0.90,3.98)	3.77 (1.47,9.65)
Diploma and above	21	36	1	1
History of Hospitalization				
Yes	46	19	3.46 (1.86,6.45)	3.95 (1.87,8.32)
No	65	93	1	1
Types of the disorder				
Depression	30	46	1	1
Bipolar	46	23	3.06 (1.55,6.05)	3.70 (1.60,8.55)
Schizophrenia	35	43	1.24 (0.61,2.36)	1.24 (0.57,2.70)
Use of Alcohols for the last one				
year				
Yes	63	41	2.27 (1.32,3.89)	1.58 (0.82,3.07)
No	48	71	1	1
Stigma				
Yes	84	67	2.09 (1.17,3.71)	2.45 (1.18,3.01)
No	27	45	1	1
Social support				
Good	27	53	1	1
Intermediate	38	43	1.73 (0.91,3.27)	1.40 (0.65,3.01)
Poor	46	16	5.64 (2.71,11.75)	3.07 (1.29,7.30)

The odds of presenting RSB among males were 2.98 (AOR = 2.98 (95% CI: 1.49, 5.95) times higher as compared to female participants. Patients who can’t read and write were four-folds higher as compared to those who were diploma/degree holders (AOR = 3.99 (95% CI: 1.53, 10.4). The odds of presenting RSB amongst participants who had a history of Hospitalization were 3.95 (AOR = 3.95 (95% CI: 1.87, 8.32) times higher than those who didn’t have. Patients with Bipolar disorder were 3.70 (A0R = 3.70 (95% CI: 1.60, 8.55)) times higher in presenting RSB compared to patients with depression. The odds of presenting RSB among participants who had perceived internal stigma were 2.45(AOR = 2.45 (95% CI: 1.18, 5.11) times higher than their counterparts. Moreover, Participants who had no social support were about 3.07 times higher than those participants who receive social support (AOR = 3.07 (95% CI, 1.29, 7.30) (Table 3).

## Discussion

The prevalence of RSB among study participants with SMD was 49.8%. This finding implies that RSB in people with SMD is an important public health concern that needs a well-designed comprehensive strategy. The magnitude of RSB founds in this study is consistent with studies conducted in Nigeria (48%) and Turkey(47.9%) [10, 48]**.** However, it’s far lower than a study conducted in Brazil (80%) [8]. This discrepancy could be attributed to the socio-demographic differences in the studies, and there might be a social desirability bias; in Ethiopia, questions related to sexuality are very sensitive. Therefore, the participants may have been prone to hiding their private issues. Likewise, this lack of precision could be associated with the study design such as sample size and measures of RSB [8].

On the other hand, the finding of this study was higher than a study conducted in the United States of America (USA) (23%) by Cary et al. [49]. The possible reason for this discrepancy could be associated with the tool difference to measure the outcome variable. In the study conducted by Cary et al., the authors applied a five-item screening tool, which is primarily designed to assess the magnitude of HIV-risk behavior. Likewise, the study wasn’t included the use of condoms during sexual activity as a variable to measure RSB. Nonetheless, this variable was included in the current study, and its use was ascertained by the ‘Yes or ‘No’ type of questions, and the majority (53.2%) of the respondents were reported, having sex without or failed to use a condom.

Regarding sex, being male has significantly increased the risk of RSB. The finding is congruent with previous studies conducted elsewhere [2, 50–53]. Having multiple sexual partners, substance use driven unprotected sexual intercourse, as well as inconsistent use of a condom is more prevalent among males [54–56]. In the Ethiopian context, males are the breadwinner, and everything including monetary assets are under their control [57, 58]. Therefore, the likelihood of having multiple sexual partners and substance use might be higher. As a result, the finding gives an insight that due attention should be given to this segment of the population.

RSB is attributed to illiterates, which is supported by a previous study [59]. This could be explained by being illiterate correlates to a lower socioeconomic status, which may impact their access to information or have limited access to health care resources and exhibit suboptimal health behaviors (i.e. disengage in preventive health care practices) [60]. Likewise, as the level of education increases, the chance of acquiring knowledge about ailments and its life-threatening complications are considered to be higher [61]. In Ethiopia, the national literacy rate is about 36% [62] and the percentage of illiteracy could be increased among PWSMD since they reported that they are discriminated against in many activities, including formal education, this could forecast a high prevalence of RSB in people with SMD [63].

Moreover, participants who were diagnosed with bipolar disorder were at higher risk of practicing RSB and this is consistent with a previous study [49]*.* As evidenced by other kinds of study, People with bipolar disorder have higher features of sexual desire, which exposes them to practice RSB [64]*.* In Ethiopia, Mental illness is a major public health problem [60]; Despite its burden, practicing RSB might have an impact on mentally ill people’s general health and may trouble day-to-day activities, create problems in the relationship, and productivity.

Perceived stigma is positively associated with RSB. This finding is also in line with other studies [27, 28, 65]. This is reasonable to say that having a perceived stigma could lead a person to develop feelings of hopelessness, worthlessness, and disregard. Besides this, perceived stigma can expose people to substance use to minimize stigma-related social, or sexual anxiety [27]. Similarly, perceived stigma results in social discrimination, which adversely prone to unstable couple relationships associated with a feeling of being rejected and de-evaluated [65]. Moreover, the Health Behavior Model, Social Cognitive Theory, and the Theory of Planned Behavior [66, 67] support that perceived internal stigma positively predicted RSB. In Ethiopia, about 50–75% of patients with mental illness perceived that they were stigmatized or had experienced some sort of stigma [68, 69]. It is a challenge for patients with SMD of recovery, upsurges RSB, and limits social and occupational functioning. As a result, the impacts of perceived stigma demand a strong effort of policymakers, local decision-makers, and other stakeholders to create awareness about it.

The study revealed that the higher odds of practicing RSB were observed among those participants who had a history of hospitalization/admission. Previous studies have witnessed the same [53, 65, 67]. And, this might be owing to the fact that people who are hospitalized are severely ill, which might aggravate the magnitude [53]. It could be notable that hospitalization might increase the health care expense of a variety of medications and absenteeism from the workplace; Therefore, it is possible to forward a recommendation that reducing the hospitalization rate could bring dual benefits; firstly, it reduces the health care cost of the individual and the government [70]. The other benefit is that it might limit the development of further sexual problems, RSB if they are treated early.

Another important finding is that participants who had poor social support experienced RSB. The result of this study is in agreement with a previous study [27]. Good social support may protect them from involving in maladaptive behaviors, as the person interact, play, relax, and communicate with friends and significant others. These could help them by normalizing symptoms of the disorder that they are suffering from [27]. Thus, strengthening or establishing good social support could refrain these people from practicing RSB; the brief strategic family therapy model also explained that to eliminate or reduce the maladaptive behaviors, such as drug use and other risk-taking behaviors, family support has a significant role [71]. Furthermore, social support is the key source of psychological health [72] and has been identified as a specific aid to accelerate recovery time [73].

Incorporating and determining other very important variables through a variety of standardized tools, for instance, social support and perceived internal stigma are the strengths of the study. In addition, recruiting participants after screening for their insight is a plus strength of the study since inviting and including participants having poor insight may affect the finding. Nevertheless, the study might not be free of some acceptable limitations. Even though some measures were applied to minimize potential biases, the study might be victimized of social desirability and recall biases; and, measuring the outcome variable (RSB) using a four-item questionnaire (considering only one “yes” among the aforementioned questionnaires to say the one had to practice RSB) which may affect the estimates and ultimately mislead the interpretation and conclusion of the study. Besides this, patients who had two and more follow-ups were recruited to the study in that the treatment could improve the mental health conditions of the patient and would refrain from acting RSB. As a result, the magnitude could be underestimated. Furthermore, it would have been more novel if the study was done by incorporating the frequency and dosage of substance use that the participants consumed. Likewise, it would have been more representative if the study was conducted in the community-based approach and complemented by qualitative studies to explore possible reasons for experiencing RSB.

## Conclusion

The result of this study showed that RSB among patients with SMD was high. Participants who were male, unable to read and write, diagnosed with bipolar disorder, had a history of hospitalization, perceived internal stigma, and poor social support was associated with presenting RSB.

Especial attention shall be given to male patients with substance users and having multiple sexual partners, as these are the pouring factors. Integrating people with SMD into adult education programs and literacy policy by approaching mental health stakeholders, as these are the deriving force. Moreover, Enduring health education regarding safe sex behavior/practice, and promoting and normalizing utilization of condoms using mass media are recommended. Likewise, building a client’s self-esteem by doing early screening in their follow-up, and link patients to clinical psychologists and engaging others for better social support systems are strongly recommended.

## Data Availability

All data generated or analysed during this study are included in this published article [and its supplementary information files].

## Abbreviations

AOR: Adjusted Odds Ratio
BPD: Bipolar Disorder
CI: Confidence Interval
COR: Crude Odds Ratio
MDD: Major Depression Disorder
OPD: Out Patient Department
OR: Odds Ratio
RSB: Risky Sexual Behavior
SMD: Severe Mental Disorder
STI: Sexual Transmitted Illness
UOG: University Of Gondar
WHO: World Health Organization
